# Anellovirus evolution during long-term chronic infection

**DOI:** 10.1093/ve/vead001

**Published:** 2023-01-05

**Authors:** Joanna Kaczorowska, Anne L Timmerman, Martin Deijs, Cormac M Kinsella, Margreet Bakker, Lia van der Hoek

**Affiliations:** Department of Medical Microbiology and Infection Prevention, Laboratory of Experimental Virology, Amsterdam UMC, University of Amsterdam, Meibergdreef 9, Amsterdam 1105 AZ, The Netherlands; Amsterdam Institute for Infection and Immunity, Postbus 22660, Amsterdam 1100 DD, The Netherlands; Department of Medical Microbiology and Infection Prevention, Laboratory of Experimental Virology, Amsterdam UMC, University of Amsterdam, Meibergdreef 9, Amsterdam 1105 AZ, The Netherlands; Amsterdam Institute for Infection and Immunity, Postbus 22660, Amsterdam 1100 DD, The Netherlands; Department of Medical Microbiology and Infection Prevention, Laboratory of Experimental Virology, Amsterdam UMC, University of Amsterdam, Meibergdreef 9, Amsterdam 1105 AZ, The Netherlands; Amsterdam Institute for Infection and Immunity, Postbus 22660, Amsterdam 1100 DD, The Netherlands; Department of Medical Microbiology and Infection Prevention, Laboratory of Experimental Virology, Amsterdam UMC, University of Amsterdam, Meibergdreef 9, Amsterdam 1105 AZ, The Netherlands; Amsterdam Institute for Infection and Immunity, Postbus 22660, Amsterdam 1100 DD, The Netherlands; Department of Medical Microbiology and Infection Prevention, Laboratory of Experimental Virology, Amsterdam UMC, University of Amsterdam, Meibergdreef 9, Amsterdam 1105 AZ, The Netherlands; Amsterdam Institute for Infection and Immunity, Postbus 22660, Amsterdam 1100 DD, The Netherlands; Department of Medical Microbiology and Infection Prevention, Laboratory of Experimental Virology, Amsterdam UMC, University of Amsterdam, Meibergdreef 9, Amsterdam 1105 AZ, The Netherlands; Amsterdam Institute for Infection and Immunity, Postbus 22660, Amsterdam 1100 DD, The Netherlands

**Keywords:** anellovirus, anellome, genetic variability, selection pressure, viral swarm, virus evolution

## Abstract

Human anelloviruses (AVs) are extremely genetically diverse, are widespread in the human population, and cause chronic infections. However, the evolutionary dynamics of AVs within single hosts is currently unknown, and it is unclear whether these changes have an implication on the long-term persistence of AVs in the host. Here, we assessed the evolutionary dynamics of six AV lineages during 30 years of chronic infection at single host resolution. The total number of substitutions and the number of variable sites increased over time. However, not all substitutions reached population fixation, showing that AV lineages form heterogeneous swarms within the host. Most substitutions occurred within a hypervariable region (HVR) located between nucleotide positions 800 and 1,300 of *ORF1*, which is known to be located within the spike domain. Different regions of the *ORF1* gene undergo either positive or negative selection pressure. Sites under strong diversifying selection pressure were detected in the HVR, while the majority of the sites under purifying selection were detected outside this region. The HVR may play the role of an immunological decoy that prevents antibodies from binding to more vulnerable parts of ORF1. Moreover, the frequent substitutions in this region may increase the chances of AV particles escaping immune recognition.

## Introduction

Anelloviruses (AVs) are small (up to 4 kb), single-stranded negative-sense circular DNA viruses belonging to the family *Anelloviridae*. Most healthy humans are infected by AVs, and the high prevalence of AVs in the human population combined with a lack of association with disease together points toward a commensal virus–host relationship (Kaczorowska and van der Hoek 2020). So far, three human-infecting genera were identified within the family *Anelloviridae: Alphatorquevirus*, *Betatorquevirus*, and *Gammatorquevirus*. All AVs share a similar genomic structure; however, the genetic diversity between lineages is considered very high (Kaczorowska and van der Hoek 2020; Arze, Springer, and Dudas et al. 2021; Varsani, Opriessnig, and Celer et al. 2021). It remains unclear what has led to the high diversity of AVs. It has been suggested, but not yet proven, that recombination events happen frequently between human-infecting AV lineages (Arze, Springer, and Dudas et al. 2021), and high rates of recombination were observed in seal (Fahsbender, Burns, and Kim et al. 2017) and felid (Kraberger, Serieys, and Richet et al. 2021) AVs. Of course, while recombination can result in novel genome variants, it is not the underlying source of genetic variation. It is most likely that the extreme diversity of AVs we observe now is an outcome of millions of years of co-evolution with hosts (Kaczorowska and van der Hoek 2020).

The mechanism of AV replication is still not fully described. AVs presumably use host polymerases with proofreading capabilities to replicate (Kakkola, Tommiska, and Boele et al. 2007); thus, we might predict that they will have a lower mutation rate than most viruses replicating with self-encoded polymerases. Still, the short generation times of the replicated AV DNAs will likely lead to higher evolutionary rates than their eukaryotic hosts. A study by Umemura and colleagues proposed quite a high (7 × 10^−4^ nucleotide substitutions per site per year) evolutionary rate of *ORF1* and *ORF2* genes of an AV (Umemura, Tanaka, and Kiyosawa et al. 2002). Another study estimated a slightly lower rate of approximately 2 × 10^−4^ nucleotide substitutions per site per year across the whole genome (Bédarida, Dussol, and Signoli et al. 2017). Both rates are high but similar to those observed in other DNA viruses like parvoviruses and plant-infecting *Geminiviridae* (Duffy, Shackelton, and Holmes 2008), both of which also rely on host polymerases. A fraction of the substitutions observed in viruses, including AVs, may be derived from host restriction editing (deamination of Cs) by apolipoprotein B mRNA-editing enzyme, catalytic polypeptide-like 3 (APOBEC3), since it has been observed occasionally in alphatorqueviruses (Ball, Curran, and Berridge et al. 1999; Tsuge, Noguchi, and Akiyama et al. 2010; Timmerman, Kaczorowska, and Deijs et al. 2022).

Viral populations in clinical samples often contain large numbers of closely related but genetically distinct variants (Domingo, Martínez-Salas, and Sobrino et al. 1985; Domingo and Perales 2019). New variants are generated continuously during virus replication, forming so-called ‘swarms’, and the relative frequency of each variant may change over time (Domingo and Perales 2019). At the moment of transmission, only a fraction of variants will cause a productive infection in the recipient, and thus, the population goes through a bottleneck event (Campo, Zhang, and Ramachandran et al. 2017). The transmitted variants will subsequently establish distinct populations in the newly infected hosts. It has been suggested that AVs form swarms in chronically infected individuals (Nishizawa, Okamoto, and Tsuda et al. 1999), but the hypothesis has yet to be explored in the datasets consisting of longitudinally collected samples.

Inter-species comparative genomic studies on AVs show variation located in the middle of the *ORF1* gene, approximately between amino acid residues 300 and 500 (i.e. nucleotides 900 and 1,500) (Nishizawa, Okamoto, and Tsuda et al. 1999; Arze, Springer, and Dudas et al. 2021). Recently, an ORF1 protein structure of the *Betatorquevirus* LY1 strain was described, showing that this hypervariable region (HVR) is mainly located in the P2 spike domain (Liou, Cohen, and Zhang et al. 2022).

It was previously shown that the AV virome (anellome) is stable and personal, and AV lineages may persist in individuals for decades (Arze, Springer, and Dudas et al. 2021; Kaczorowska, Deijs, and Klein et al. 2022). The current study looked at the heterogeneity and evolution of six AV lineages in healthy individuals followed up for more than 30 years. We assessed the evolutionary patterns generated in *ORF1* sequences and identified substitution hotspots. We evaluated whether novel variants replaced ancestors or whether they coexisted in the host. Finally, we analyzed whether AV sequences displayed evidence of positive or negative selection pressure. The results shed more light on the evolutionary dynamics of AVs at single host resolution.

## Materials and methods

### Sequence data

Illumina reads derived from the longitudinally sampled serum of two healthy male subjects (subject 1 and subject 2) were obtained from National Center for Biotechnology Information (NCBI) BioProject PRJNA785545, and the data analysis was performed as described previously (Kaczorowska, Deijs, and Klein et al. 2022). Both subjects were participants of Amsterdam Cohort Studies on human immunodeficiency virus (HIV) infection and acquired immunodeficiency syndrome, which consist of men having sex with men living in the Amsterdam area. Samples were collected every few months for 413 (subject 1) and 386 months (subject 2). Subject 1 was 41 years old and subject 2 was 35 years old at the beginning of the follow-up. The sampling was temporarily suspended between 3 December 1996 and 10 April 2003 (subject age 51.5–58.9 years) for subject 1 and between 16 December and 7 April for subject 2 (age 44.3–50.6 years), resulting in lacking samples in these periods.

Briefly, quality-trimmed reads were *de novo* assembled using SPAdes (using the -careful setting) and identified as AVs using the Cenote-Taker2 virus discovery pipeline (Tisza, Pastrana, and Welch et al. 2020). The contigs from all time points were then clustered per subject in Codon Code aligner version 8.0.2, using a 95 per cent identity threshold.

### Lineage and time point selection

We selected six chronically occurring lineages that were previously included in a lineage-specific quantitative polymerase chain reaction (qPCR) assay (Kaczorowska, Deijs, and Klein et al. 2022). The availability of qPCR data allowed us to select samples with sufficient DNA copy numbers (>500 copies per milliliter). We designated a reference sequence for each lineage, which was the contig derived from the earliest possible time point. The *ORF1* nucleic acid sequences were extracted from all reference sequences using EMBOSS getorf (Rice, Longden, and Bleasby 2000). We decided to use solely *ORF1* sequences because (1) we observed that all or the vast majority of nucleotide variations were located within this gene and (2) the region of *ORF1* was fully represented at sufficient sequencing depth (at least 1,000 mapped reads) for all reference sequences of the selected lineages.

To identify the possible variants in the selected time points, quality-trimmed paired reads were mapped to the reference sequences of all lineages using Burrows-Wheeler Aligner (Li and Durbin 2009). The resulting binary alignment map files were indexed, and the duplicates were marked and removed using Genome Analysis Toolkit (GATK) (van der Auwera and O’Connor 2020). Outputs were then analyzed with the variant caller LoFreq (Wilm, Ppk, and Bertrand et al. 2012). The variant call format (VCF) outputs were filtered using an allele frequency (AF) threshold of >0.05 (5 per cent) and a quality threshold of 100. Filtered outputs were converted into a table using GATK and imported into R (version 4.1.3) for further analysis. All steps except R analysis were performed on the Lisa high-performance computing cluster (surfsara.nl).

For each lineage, we selected time points for further analysis using the following criteria: (1) at least 500 DNA copies per milliliter serum (based on qPCR data); (2) at least 1,000 reads mapping to the reference *ORF1* sequence; (3) more than 90 per cent coverage of the reference *ORF1* sequence; and (4) absence of putative APOBEC3 editing. We considered the sequences to be APOBEC3 edited if 5 per cent or more of the guanines were substituted by adenines on the coding strand when compared to the corresponding reference sequence. All the lineages and the time points are shown in Supplementary Table S1, and the output of the variant-calling analysis is reported in Supplementary Table S2.

### Identification of the HVR

To define the location of the HVR, we counted the number of variants at each nucleotide position of the *ORF1* gene and plotted them as a histogram. Next, we obtained the consensus sequence of each lineage at each selected time point using the VCF-consensus script within VCFtools, and the sequences were arranged temporally. The number of substitutions compared to the reference sequence was estimated using MEGAX (Kumar, Stecher, and Li et al. 2018). The number of synonymous and non-synonymous substitutions across *ORF1* was estimated in MEGAX using the Nei-Gojobori method (Nei and Gojobori 1986; Kumar, Stecher, and Li et al. 2018). The statistical significance was estimated using Wilcoxon sum-rank test.

### Accumulation of substitutions

Next, we evaluated whether the number of variable positions compared to the reference sequence increased over time. We grouped the VCF-derived table by the subjects’ ages and by lineage and counted the number of substitutions. Spearman’s rank correlation test was performed in R using the stats package.

### Selection pressure analysis

To evaluate whether individual sites within the *ORF1* gene have experienced selection pressure or evolved neutrally, we applied three methods for inferring selection pressure: Fixed Effects Likelihood (FEL), Single-Likelihood Ancestor Counting (SLAC) (Kosakovsky Pond and Frost 2005), and Mixed Effects Model of Evolution (MEME) (Murrell, Wertheim, and Moola et al. 2012). First, we removed the single-time occurring variants from the VCF files and applied the 0.05 AF filter using GATK VariantFiltration and SelectVariants commands. Consensus sequences of the filtrated VCF were obtained using the VCF-consensus script within VCFtools. The maximum-likelihood phylogenetic trees of each lineage were obtained using MEGAX (Kumar, Stecher, and Li et al. 2018). SLAC, FEL, and MEME analyses were performed using a command-line version of Hyphy (Kosakovsky Pond, Poon, and Velazquez et al. 2020).

To show locations of codons under positive and negative selection pressure, we aligned ORF1 amino acid sequences with the *Betatorquevirus* strain LY1 (accession number YP_007518450.1) and *Alphatorquevirus* strain JA20 (AAD44681.1). The locations of protein domains of LY1 and JA20 have been defined previously (Liou, Cohen, and Zhang et al. 2022); thus, we could annotate domains in our lineages using these as references.

## Results

### Identification of the HVR

**Figure 1.**
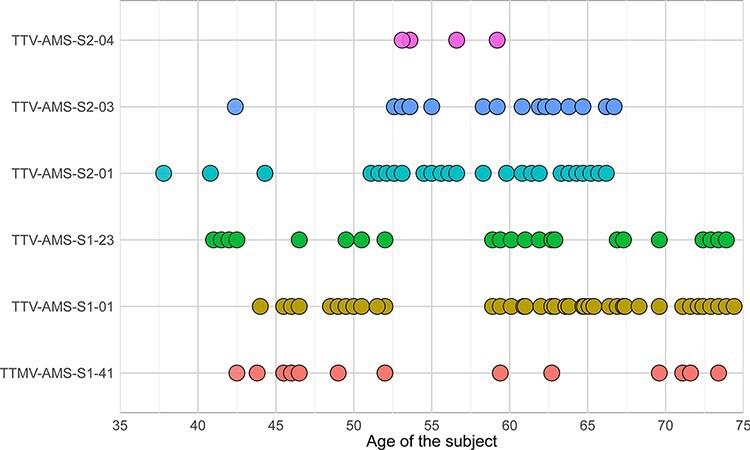
The number of time points included in the analysis for each lineage and the age of the subjects.

We used an existing blood metagenomics dataset obtained from two healthy male subjects (Kaczorowska, Deijs, and Klein et al. 2022) to study the evolution of six chronic AV lineages—five alphatorqueviruses (torque teno virus; TTV) and one betatorquevirus (torque teno mini virus, TTMV; Fig. 1). The time points were selected based on the copy number, coverage, and absence of putative editing by APOBEC3 (the list of samples is presented in Supplementary Table S1). The length of viral persistence ranged between 6.1 (TTV-AMS-S2-04) and 32.9 years (TTV-AMS-S1-23; Fig. 1).

**Figure 2.**
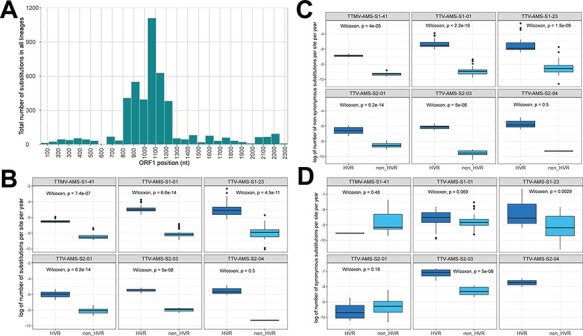
Estimation of the HVR. (A) Histogram of the total number of substitutions at each ORF1 position. The logarithm of (B) the total number of substitutions per site and per year, (C) non-synonymous substitutions, (D) and synonymous substitutions, for each selected lineage, within and outside the HVR. The number of substitutions was calculated by comparing the reference genome (the first time point) with the other time points. The *P*-values (estimated using Wilcoxon sum-rank test) are shown on the top of the boxplots.

Most substitutions were found between nucleotide (nt) positions 800 and 1,300 of the *ORF1* gene (Fig. 2A), corresponding to the previously identified HVR. To statistically compare the variability of the HVR with the rest of *ORF1*, we estimated the total number of variants in each time point, with the first time point used as a reference sequence. The total number of substitutions per site per year was significantly higher within the 800–1,300 nt HVR compared to the remainder of *ORF1* in five out of six lineages (Fig. 2B). Furthermore, a significantly higher number of non-synonymous substitutions were present in the HVR (*P*-values <0.05 for five out of six lineages; Wilcoxon sum-rank test; Fig. 2C). When comparing the synonymous sites, significant differences along *ORF1* were found in only two lineages—TTV-AMS-S1-23 and TTV-AMS-S2-03 (*P*-value <0.05; Fig. 2D).

### Accumulation of substitutions

The variant-calling analysis resulted in the following total counts of substitutions across the whole follow-up: 132 for TTMV-AMS-S1-41, 2,204 for TTV-AMS-S1-01, 1,031 for TTV-AMS-S1-23, 600 for TTV-AMS-S2-01, 521 for TTV-AMS-S2-03, and 15 for TTV-AMS-S2-04 (Supplementary Table S2). The total number of variable sites (the unique sites within *ORF1* that showed variants) ranged between 7 (TTV-AMS-S2-04) and 152 (TTV-AMS-S1-01; Supplementary Fig. S1). The lineages that were present in the subjects for >20 years tended to have more total substitutions and more variable sites; however, TTMV-AMS-S1-41 (present in the subject for 31.4 years) showed a much lower (132) number of accumulated substitutions compared to TTV-AMS-S1-01 (2,204) and TTV-AMS-S1-23 (1,031; present for 30.4 and 32.9 years, respectively).

**Figure 3.**
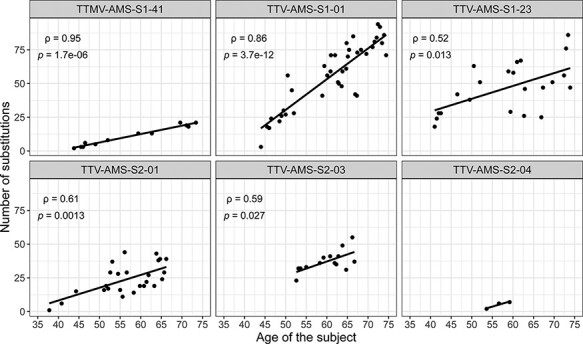
The accumulation of substitutions over time. The relationship between the number of substitutions compared to the reference (first time point of the follow-up) and the age of the subjects. The rho (ρ) and *P*-values were estimated using Spearman’s rank correlation test. No statistical test was performed for TTV-AMS-S2-04 due to a low number of samples (three time points). The best-fit lines were generated using the linear model.

To test whether the number of mutated sites correlated with the follow-up time, we performed a Spearman’s rank correlation coefficient test (Fig. 3; Supplementary Table S3). The number of variants increased over years for all lineages, and the correlation was significant for five of the lineages (TTV-AMS-S2-04 was excluded from statistical analysis due to a low number of time points). The most significant correlation was observed for TTV-AMS-S1-01 (*P*-value = 3.7 × 10^–12^). In this lineage, the last time point was only 97.55 per cent identical to the reference (seventy-one substitutions). Importantly, there was no significant relationship between the number of copies or reads per million and the number of substitutions observed (Supplementary Fig. S2); therefore, we can exclude that the variation we found per lineage was influenced by fluctuation in viral load.

### Heterogeneity analyses

**Figure 4.**
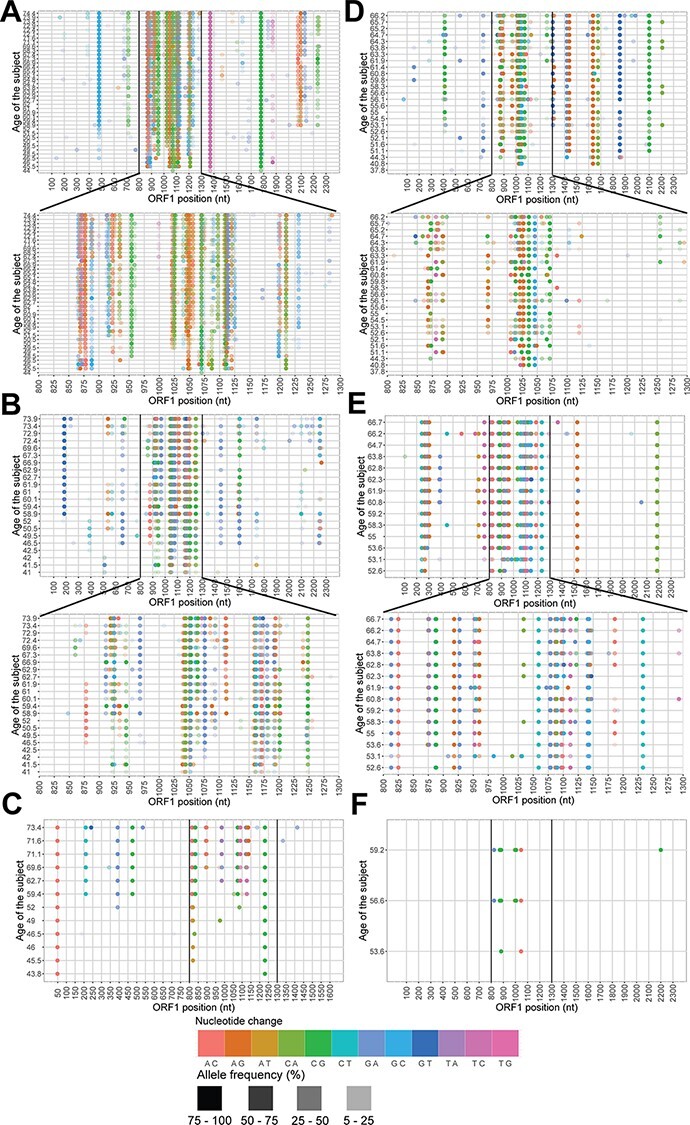
Heterogeneity of lineage populations in time. Nucleotide changes in time for (A) TTV-AMS-S1-01, (B) TTV-AMS-S1-23, (C) TTMV-AMS-S1-41, (D) TTV-AMS-S2-01, (E) TTV-AMS-S2-03, and (F) TTV-AMS-S2-04. In each panel, the upper part shows the whole ORF1 length, while the lower part (if present) shows only the hypervariable region. Different colors represent different nucleotide changes, and the strength of the color represents the frequency of the variant.

We hypothesized that the lineage populations are heterogeneous and form swarms. We visualized all the changes in nucleotides across the follow-up period for each lineage (Fig. 4). We observed three types of substitutions: (1) those that are introduced and then persist until the end of the follow-up (observed in all lineages); (2) those that are introduced but then disappear in the next time point(s) and sometimes re-appear again (observed in all lineages except TTMV-AMS-S1-41 and TTV-AMS-S2-04); (3) some that are introduced in one time point but then remain undetected until the end of the follow-up (present in all lineages; the variants detected in only one time point are listed in Supplementary Table S4). From this, we conclude that the newly introduced variants do not always replace the ancestors but coexist throughout the follow-up as a swarm.

Three out of six tested lineages (TTV-AMS-S1-01, TTV-AMS-S1-23, and TTV-AMS-S2-01) showed variants in the first (reference) time point (Supplementary Table S5), which may mean that these lineages were present in a form of a swarm already in their first time point.

### Selection pressure

There was a higher number of non-synonymous substitutions than synonymous substitutions within the *ORF1* gene, and thus, we hypothesized that it experiences positive (diversifying) selection pressure. It is important to mention that, for this analysis, we needed to first obtain consensus sequences from each of the time points. Therefore, we had to assume that the substitutions are fixed in the subsequent temporal samples, even though the data shown in the previous section suggested otherwise (Fig. 4). We minimalized the effect of lack of fixation by removing the 166 variants that were present in only one time point (Supplementary Table S4). We performed three statistical tests for selection pressure evaluation: FEL, MEME, and SLAC. First, the average ratio of non-synonymous substitutions per non-synonymous site (dN) and synonyms substitutions per synonymous site (dS), dN/dS, was calculated for all lineages using SLAC (Supplementary Table S6). Five out of six lineages showed dN/dS >1; however, for most of the lineages, the value was not significantly above 1, which suggests a weak positive selection pressure or that the lineages evolve neutrally. Only TTV-AMS-S2-01 showed the dN/dS = 3.5, which suggests a strong positive selection. Interestingly, one lineage, TTV-AMS-S2-03, showed dN/dS = 0.89, which may mean a weak negative selection pressure.

**Figure 5.**
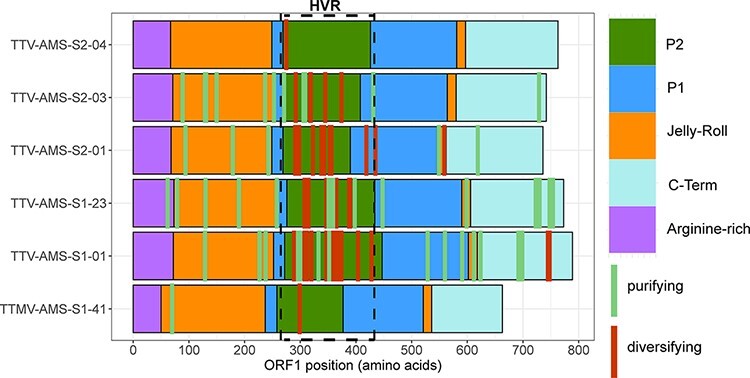
Selection pressure in the ORF1 protein. The location of the hypervariable region is indicated with a dashed-line box. The approximate locations of the protein domains were defined based on the results of Liou, Cohen, and Zhang et al. (2022).

The number of sites found to be under selection pressure varied depending on the method used (Supplementary Table S7). We found between one and eighteen sites under positive selection pressure detected with at least one of the three methods. The majority (between 82 per cent and 100 per cent depending on the lineage) of the sites under significant positive selection were found in HVR (Supplementary Table S7; Fig. 5).

Significant negative selection was found in all but one (TTV-AMS-S2-04) lineage. The highest number of purifying selection sites was detected in TTV-AMS-S1-23 (seventeen codons). The majority (between 64 per cent and 100 per cent, depending on the lineage) of the codons under purifying selection pressure were detected outside the HVR (Supplementary Tables S7 and S8; Fig. 5). These codons were almost equally present upstream (mainly within the putative jelly-roll domain) and downstream (C-terminal domain) of the HVR. There were almost no sites under selection pressure detected within the P1 and arginine-rich domains (Fig. 5).

## Discussion

In this study, we assessed the heterogeneity and evolution of six AV lineages chronically infecting two individuals. We show that AVs evolve in the subjects—the number of substitutions increased over time for all tested lineages. Strikingly, the substitutions were not always fixed in the lineage genome, suggesting that new variants do not always replace the ancestors but coexist instead. In three lineages (TTV-AMS-S1-01, TTV-AMS-S1-23, and TTV-AMS-S2-01), we observed polymorphic sites (i.e. sites with more than one possible variant) already in the first time point, which may mean that they were present as swarms already at the beginning of the follow-up. The uncovered pattern of evolution suggests an adaptive response to host antiviral defenses, and the formation and expansion of swarms over time may be a strategy for avoiding host immunity.

The number of substitutions did not always increase at the same rate. There were roughly eight to eighteen times fewer substitutions generated in the only betatorquevirus in the lineage selection (TTMV-AMS-S1-41) compared to the remaining two lineages from the subject, even though the follow-up period of all three lineages was similar (more than 30 years). In this study, it was not feasible to include more *Betatorquevirus* lineage because there were only three persistent betatorqueviruses in the tested subjects (Kaczorowska, Deijs, and Klein et al. 2022), and only one of these three (TTMV-AMS-S1-41) showed sufficient DNA copy numbers in more than one time point. It is possible that betatorqueviruses evolve at a lower rate than alphatorqueviruses, but an evolution study involving more *Betatorquevirus* lineages should be performed to support this claim.

The majority of substitutions of *ORF1* were located between 800 and 1,300 nucleotides. This region coincides with the HVR as described by Arze, Springer, and Dudas et al. (2021) (900 and 1,500 nucleotides), which was established based on 2,101 lineages obtained in their study, plus NCBI databases (Arze, Springer, and Dudas et al. 2021). We therefore confirmed the most variable region of AVs and showed its evolutionary dynamics at single host resolution.

Across the whole *ORF1* gene, the dN/dS ratio was just slightly higher than 1 for four out of six lineages, and one lineage showed dN/dS of 3.5. This suggests that the complete *ORF1* gene is under only a slight diversifying selection pressure or evolves neutrally. Only one of the lineages, TTV-AMS-S2-03, showed a dN/dS of <1, suggesting a weak purifying selection. For five out of six lineages, there were significantly more non-synonymous substitutions within the HVR than outside this region. It was therefore no surprise that the majority of sites undergoing positive selection were also located in HVR (located within the putative P2 spike domain; Liou, Cohen, and Zhang et al. 2022). On the other hand, the sites under purifying selection pressure were mainly present outside HVR—both upstream and downstream. These regions are part of the 3D structure of the ORF1 protein—putative jelly-roll and C-terminal domain, the domains involved in such essential processes as, respectively, DNA binding and particle assembly (Liou, Cohen, and Zhang et al. 2022). We detected no sites under purifying selection pressure in TTV-AMS-S2-04 lineage, which was probably due to a low number of time points and variants of the lineage. Unsurprisingly, the lineages with the highest numbers of variants showed also the highest numbers of sites under negative selection. There were almost no sites under selection pressure detected in the P1 spike region, which is hypothesized to be involved in the receptor binding, with high levels of conservation. The high level of amino acid conservation we also found in our study implies an important role in protein function that makes sense in the context of the recently published AV structure (Liou, Cohen, and Zhang et al. 2022).

The frequent substitutions in HVR may attenuate the binding of antibodies, enabling the virus to escape host immunity. ORF1 protein elicits immune responses (Kakkola, Bondén, and Hedman et al. 2008) although they may not be very strong, especially in the case of alphatorqueviruses (Venkataraman, Swaminathan, and Arze et al. 2022). The most immunogenic region is located at the C-terminal of the ORF1 protein, and HVR was reported to elicit weaker responses (Venkataraman, Swaminathan, and Arze et al. 2022). However, it is important to mention that Venkataraman et al. studied linear epitopes only, and the immune response targeting the supposedly heavily structured HVR may have been missed in the assay (Liou, Cohen, and Zhang et al. 2022; Venkataraman, Swaminathan, and Arze et al. 2022). Thus, more research is needed to fully confirm the immunogenicity of various ORF1 regions.

It was shown that the hypervariable epitopes are largely exposed, which led to a hypothesis that these epitopes may act as immunological decoys and in this way prevent the recognition of conserved capsid regions, e.g. the ones involved in the entry into the host cell (Liou, Cohen, and Zhang et al. 2022). Such a strategy was described before in HIV-1 (Moore, Crooks, and Porter et al. 2006) and hepatitis C virus (Mondelli, Cerino, and Segagni et al. 2001; Keck, Girard-Blanc, and Wang et al. 2016). It is considered less likely that the HVR of AV is involved in the receptor binding or connected with the presumed ability of AVs to infect a variety of cell types (Liou, Cohen, and Zhang et al. 2022). More research on the compartmentalization of infection and virus tropism is needed to shed more light on the importance of the HVR in AV infection.

## Supplementary Material

vead001_Supp

## Data Availability

The raw Illumina sequencing reads are available under the NCBI BioProject number PRJNA785545. All the scripts used in this study and R code are available in a GitHub repository under the following link: https://github.com/joannakaczorowska/anellovirus_evolution/.
