# Supplementary material for: Anellovirus evolution during long-term chronic infection
Source: Virus Evol. 2023 Jan 5;9(1):vead001. doi: 10.1093/ve/vead001 (PMC9885978; doi:10.1093/ve/vead001)
Supplement: vead001_Supp [file vead001_supp.zip › Supplementary Figures_14-Dec-22.docx]

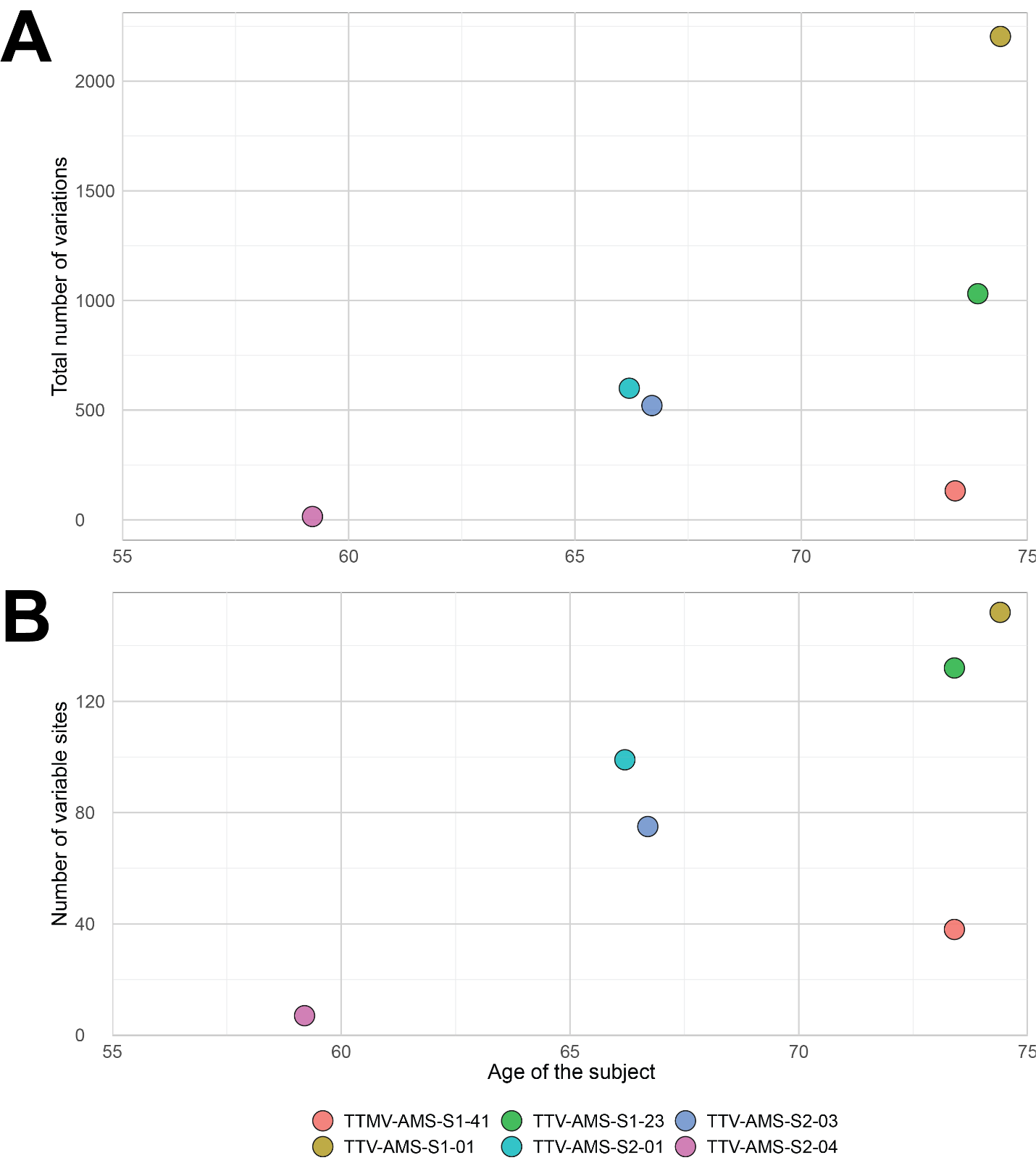


**Figure S1. Relationship between the total number of variants (A) or the number of variable sites (B) and the age of the subjects.**

**
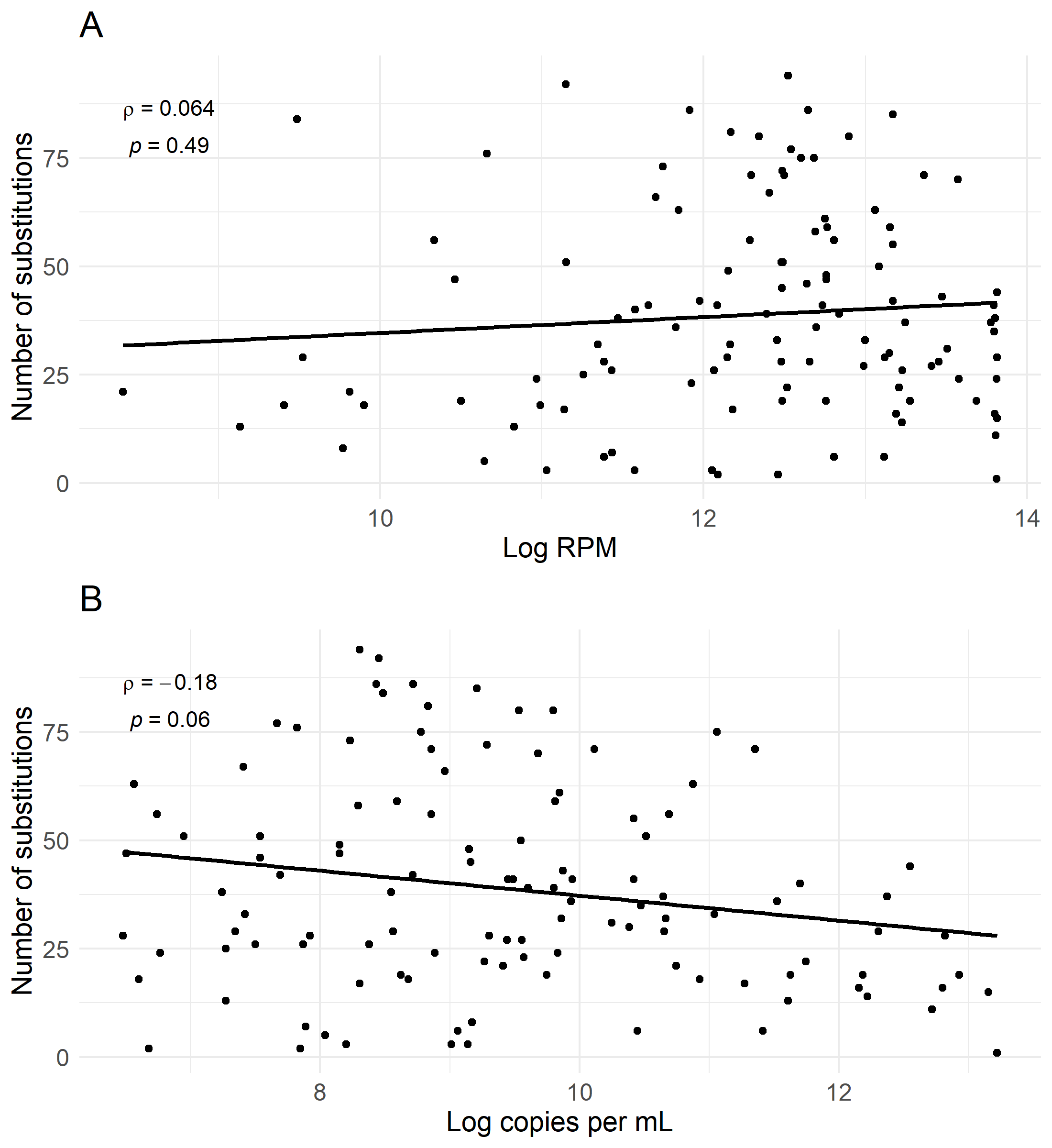
**

**Figure S2.** A correlation between the logarithm of reads per million (RPM; A) or logarithm copies per ml (B) and number of detected mutations. Neither relationships were significant: A) p-value = 0.49, rho = 0.064; B) p-value = 0.06, rho = -0.18.
